# Continuous Renal Replacement Therapy Guided by Bedside Ultrasound in an Extremely Low Birth Weight Infant

**DOI:** 10.1007/s12098-023-05015-8

**Published:** 2024-01-10

**Authors:** Guoliang Xiao, Shangjie Xiao, Yue Wang, Chun Shuai, Chuan Nie

**Affiliations:** 1 Department of Neonatology/National Key Clinical Specialty Construction Project, Guangdong Women and Children Hospital, Guangzhou, 511442 Guangdong China; 2 Guangdong Neonatal ICU Medical Quality Control Center, Guangzhou, 511442 Guangdong China; 3 Department of Neonatal Surgery, Guangdong Women and Children Hospital, Guangdong, China

*To the Editor:* Continuous renal replacement therapy (CRRT) remains a significant challenge for extremely low birth weight infants [1, 2]. Only 0.7% of neonates with acute kidney injury (AKI) have the opportunity for CRRT [3]. The survival rate of CRRT has been reported as only 31% in neonates weighing <3 kg [4]. Here we report CRRT guided by bedside ultrasound in an extremely preterm infant.

A male infant with a gestational age of 24 wk and a birth weight of 640 g had neonatal necrotizing enterocolitis (NEC) on the 11th day, with postoperative onset of AKI. The decision to perform CRRT was made after obtaining ethical approval and parental consent on day 22, when the infant weighed 720 g.

The infant developed hypotension during CRRT, and bedside ultrasonography revealed that the entire heart was smaller, with a left ventricular ejection fraction (LVEF) of 60%. Simultaneous measurements of systolic flow velocity and diastolic flow velocity in the middle cerebral artery were 0.36 m/s and 0.04 m/s, and resistance index was 0.88. These findings indicated insufficient blood volume, resulting in cardiac insufficiency and a subsequent decrease in cerebral blood flow.

Accordingly, fluid resuscitation therapy was administered following the protocol for patients with severe anemia in small amounts of red blood cell infusion, albumin supplementation, and plasma. Catecholamines were used simultaneously to maintain blood pressure stability. Fluid therapy guided by hemodynamic screening ultimately stabilized the blood pressure. Repeat screening revealed normal cardiac function. However, the blood flow velocity in the middle cerebral artery did not significantly improve. CRRT was administered for 31 h, followed by ultrafiltration 74.6 ml. However, the infant eventually died of pulmonary hypertension.

To the best of our knowledge, hemodynamic assessment guided by bedside ultrasonography can better respond to hypotension, shock, and intracranial hemorrhage during CRRT.

## References

[1] Noh ES, Kim HH, Kim HS (2019). Continuous renal replacement therapy in preterm infants. Yonsei Med J.

[2] Starr MC, Charlton JR, Guillet R (2021). Advances in neonatal acute kidney injury. Pediatrics.

[3] Jetton JG, Boohaker LJ, Sethi SK (2017). Incidence and outcomes of neonatal acute kidney injury (AWAKEN): a multicentre, multinational, observational cohort study. Lancet Child Adolesc Health.

[4] Garzotto F, Vidal E, Ricci Z (2020). Continuous kidney replacement therapy in critically ill neonates and infants: a retrospective analysis of clinical results with a dedicated device. Pediatr Nephrol.

